# Supplementary material for: Direct Detection of Glutathione Biosynthesis, Conjugation, Depletion and Recovery in Intact Hepatoma Cells
Source: Int J Mol Sci. 2022 Apr 25;23(9):4733. doi: 10.3390/ijms23094733 (PMC9104575; doi:10.3390/ijms23094733)
Supplement: Supplementary file 1 [file ijms-23-04733-s001.zip › ijms-1672203-supplementary.pdf]

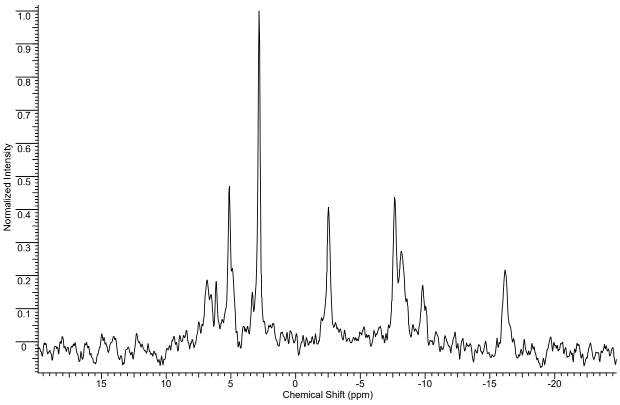

Figure S1. ^{31}P NMR spectrum of perfused JM-1 cells obtained just prior to mBBr addition.

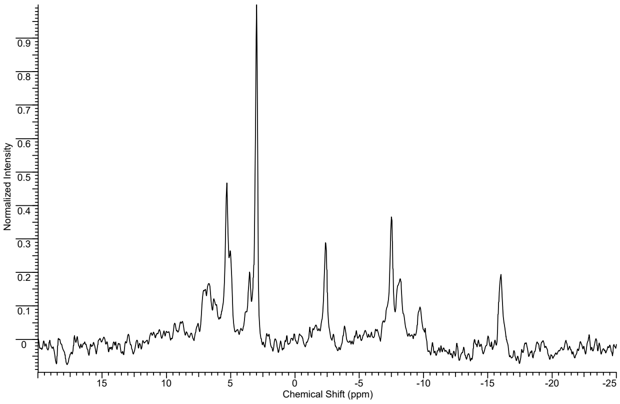

Figure S2. ^{31}P NMR spectrum of perfused JM-1 cells obtained 4 h after mBBr addition.

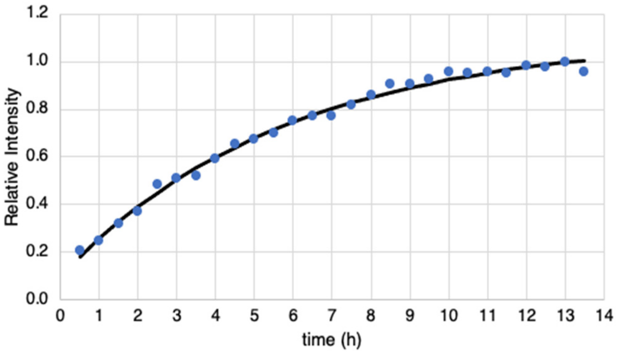

Figure S3. Time course of the ^{13}C-GSH peak in JM-1 cells after switching to media containing [3,3′-$^{13}C_2$]-cystine. 30 min acquisitions. Exponential fit to the data with $k = 0.172\ h^{-1}$.

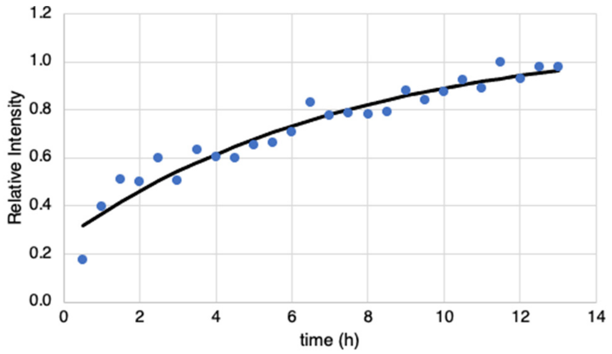

Figure S4. Time course of the ^{13}C-GSH peak in JM-1 cells after switching to media containing [3,3′-$^{13}C_2$]-cystine. 30 min acquisitions. Exponential fit to the data with k = 0.133 h^{-1}.

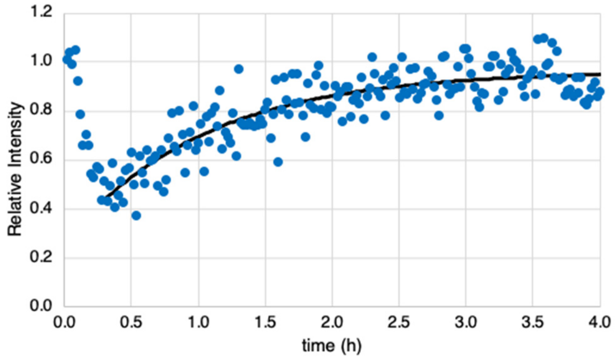

Figure S5. Time course of the ^{13}C-GSH peak in JM-1 cells during and after addition of mBBr to the perfusate. 1.2 min acquisitions. Exponential fit to the recovery data with k = 0.961 h^{-1}.

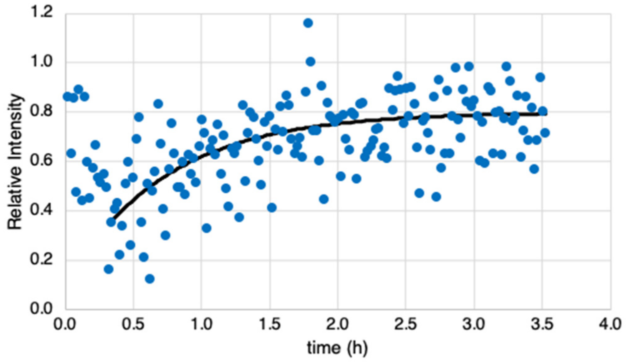

Figure S6. Time course of the ^{13}C-GSH peak in JM-1 cells during and after addition of mBBr to the perfusate. 1.2 min acquisitions. Exponential fit to the recovery data with k = 1.28 h^{-1}.
